# Triglyceride to High-Density Lipoprotein Cholesterol Ratio Predicts Cardiovascular Outcomes in Prevalent Dialysis Patients

**DOI:** 10.1097/MD.0000000000000619

**Published:** 2015-03-13

**Authors:** Hung-Yuan Chen, Wan-Chuan Tsai, Yen-Ling Chiu, Shih-Ping Hsu, Mei-Fen Pai, Ju-Yeh Yang, Yu-Sen Peng

**Affiliations:** From the Division of Nephrology (H-YC, W-CT, Y-LC, S-PH, M-FP, J-YY, Y-SP), Department of Internal Medicine, Far Eastern Memorial Hospital, New Taipei City; and Division of Nephrology (H-YC, Y-LC, S-PH, M-FP, J-YY, Y-SP), Department of Internal Medicine, National Taiwan University Hospital, National Taiwan University College of Medicine, Taipei, Taiwan.

## Abstract

Triglyceride to high-density lipoprotein cholesterol (TG/HDL-C) ratio, an indicator of atherogenic dyslipidemia, is a predictor of cardiovascular (CV) outcomes in the general population and has been correlated with atherosclerotic events. Whether the TG/HDL-C ratio can predict CV outcomes and survival in dialysis patients is unknown.

We performed this prospective, observational cohort study and enrolled 602 dialysis patients (539 hemodialysis and 63 peritoneal dialysis) from a single center in Taiwan followed up for a median of 3.9 years. The outcomes were the occurrence of CV events, CV death, and all-cause mortality during follow-up. The association of baseline TG/HDL-C ratio with outcomes was explored with Cox regression models, which were adjusted for demographic parameters and inflammatory/nutritional markers.

Overall, 203 of the patients experienced CV events and 169 patients died, of whom 104 died due to CV events. Two hundred fifty-four patients reached the composite CV outcome. Patients with higher TG/HDL-C levels (quintile 5) had a higher incidence of CV events (adjusted hazard ratio [HR] 2.03, 95% confidence interval [CI] 1.19–3.47), CV mortality (adjusted HR 1.91, 95% CI 1.07–3.99), composite CV outcome (adjusted HR 2.2, 95% CI 1.37–3.55), and all-cause mortality (adjusted HR 1.94, 95% CI 1.1–3.39) compared with the patients in quintile 1. However, in diabetic dialysis patients, the TG/HDL-C ratio did not predict the outcomes.

The TG/HDL-C ratio is a reliable and easily accessible predictor to evaluate CV outcomes and survival in prevalent nondiabetic dialysis patients.

ClinicalTrials.gov: NCT01457625

## INTRODUCTION

Atherogenic dyslipidemia combined with high triglyceride (TG) and low high-density lipoprotein cholesterol (HDL-C) levels have been reported to strongly predict cardiovascular (CV) morbidity, and especially coronary artery disease in the general population.^1,2^ In addition, to subjects with stroke or transient ischemic attack, the presence of atherogenic dyslipidemia predicts the occurrence of CV events even if they are under adequate 3-hydroxy-3-methylglutaryl-coenzyme A reductase inhibitor (statin) therapy.^3^ Several investigations performed on the general population have also shown a correlation between the TG to HDL-C (TG/HDL-C) ratio and the concentration of low-density lipoprotein cholesterol (LDL-C) type B, severity of insulin resistance, and coronary atherosclerotic lesions.^4–6^ The TG/HDL-C ratio has been shown to precisely predict the occurrence of myocardial infarction, ischemic heart disease,^7,8^ and CV mortality, both in women with coronary artery disease and in the general population.^9,10^ Therefore, in the era of aggressive LDL-C reduction with statins, scientific evidence implicates low HDL-C and high TG as the residual CV risk observed after LDL-C has been lowered. Several landmark trials attempted to modulate the TG/HDL-C ratio in dyslipidemic patients by using fenofibrate, torcetrapib, or niacin; however, they did not clearly reduce the risk of CV events.^11–13^

In patients with chronic kidney disease (CKD), LDL-C is not a good marker to assess the risk of CV events owing to its weak association with coronary events in CKD patients with a low estimated glomerular filtration rate.^14,15^ In addition, in trials performed in dialysis patients, no CV benefits have been clearly demonstrated with aggressive LDL-C reduction strategies.^16,17^ Interestingly, hypertriglyceridemia, the dominant dyslipidemia in CKD patients, has been reported to be an inverse predictor of mortality in nondialyzed CKD patients^18^ but to have a U-shaped relationship with mortality in hemodialysis (HD) patients.^19,20^ In contrast, HDL-C has been reported to have a discordant association with all-cause or CV mortality in HD patients.^21,22^ Whether the TG/HDL-C ratio is a potential or an even better predictor of CV outcomes in dialysis patients remains unknown because of the limited amount of data on the prognostic utility of the TG/HDL-C ratio in this population. Therefore, we initiated this prospective, observational study to determine whether the TG/HDL-C ratio can predict CV events and CV mortality in prevalent dialysis patients.

## MATERIALS AND METHODS

### Subjects

This was a prospective study performed using 3 pooled patient cohorts. The first cohort was composed of 370 prevalent HD patients, the second cohort was composed of 238 prevalent HD patients, and the third cohort was composed of 220 HD patients and 63 peritoneal dialysis (PD) patients. These patient cohorts have been described previously in more detail.^23–25^ In brief, the 3 cohorts were collected prospectively and intentionally to investigate the associations among serum high-sensitivity C-reactive protein (hs-CRP), fetuin A, inflammatory markers, and visceral adiposity with specific outcomes (such as arteriovenous access patency and CV events) in prevalent dialysis patients at the Far Eastern Memorial Hospital (FEMH) from 2007 to 2011. In the 3 cohorts, the patients with available data on TG and HDL-C levels were included in the analysis, and the exclusion criteria for all 3 cohorts were as follows: active infection, recent hospitalization within 3 months, psychotic illness or other communication problem, active malignancy, <20 years, and receiving HD or PD for <3 months. All of the subjects provided written informed consent, and the ethics committee of FEMH approved the study protocol (www.ClinicalTrials.gov, NCT01457625). In total, 602 patients (age 60±12 years, 304 women) who underwent prevalent HD (539) and PD (63) at the FEMH, Taiwan, were enrolled from February 2007 (first cohort), March 2009 (second cohort), and March 2011 (third cohort). The enrolled HD patients underwent 3.5 to 5 hours of HD with bicarbonate dialysate 3 times a week, and the enrolled PD patients received conventional glucose-based lactate buffer PD solutions (Ultrabag; Baxter, Singapore) and/or once-daily icodextrin-based solution (Extraneal; Baxter, Singapore) for a total of 8 to 12 L, 3 to 5 exchanges per day. The median dialysis vintage before recruitment was 3.6 years (range 0.4–25.4 years).

### Measurements of Clinical Parameters and Nutritional Status

Demographic data, a concurrent medical history of CV disease defined as previous stroke, coronary artery disease (including myocardial infarction and unstable angina), decompensated heart failure, and smoking status were recorded. Venous blood was sampled in the morning after an overnight fast of >8 hours before the patient's midweek dialysis session in the HD patients or before the first daily dwell of dialysate in the PD patients. Whole blood samples were obtained for measurements of hemoglobin, and serum for creatinine, calcium, phosphorus, potassium, uric acid, albumin, TG, total cholesterol (T-CHO), LDL-C, and HDL-C levels. Intact parathyroid hormone levels were determined by immunoassay.

The nutritional status of the participants was quantified using the geriatric nutritional risk index (GNRI), which was calculated based on serum albumin level and body weight as follows: GNRI = [14.89 × albumin level (g/dL)] + [41.7 × body weight/WLo], where WLo is the ideal body weight calculated from the Lorentz equation. The GNRI has previously been validated in dialysis patients, and a higher GNRI indicates better nutritional status.^26^ The hs-CRP levels were determined using the immunonephelometric method using a Tina-quant CRP (Latex) ultrasensitive assay (D & P Modular Analyzer; Roche Diagnostics GmbH, Mannheim, Germany).

### Outcomes

The primary outcomes were CV events and CV mortality, considered either separately or jointly (composite CV outcome). CV events were defined as the new occurrence of CV events, including coronary events (nonfatal myocardial infarction, unstable angina, and coronary revascularization), hospitalized heart failure, hospitalized incident stroke (either ischemic or hemorrhagic stroke), and incident peripheral arterial occlusion disease requiring surgical intervention. CV mortality was defined as death caused by the CV events defined above. The secondary outcome was defined as all-cause mortality. The outcome information was centrally adjudicated, in accordance with prespecified definitions, by trained clinicians and nephrologists.

Follow-up was started from February 2007 (first cohort entry), March 2009 (second cohort entry), or March 2011 (third cohort entry) and censored on the date of the first CV event, at the end of the study (July 1, 2014), on the date of death or renal transplantation, or at the time the patients were transferred to other dialysis facilities and were no longer followed up, whichever came first.

### Statistical Analysis

Continuous data were presented as mean ± SD or median (interquartile range), and categorical data were reported as percentages. Differences in baseline characteristics and biochemical parameters between the HD and PD patients were compared using the Student *t* test and Mann–Whitney *U* test. Comparisons of the patients in different quintiles of TG/HDL-C ratio were performed by analysis of variancefor continuous variables and the nonparametric Kruskal–Wallis test for nonnormally distributed continuous variables. The χ^2^ test was used for categorical variables.

Outcome analysis was performed using Cox proportional hazard models. We used the “Enter” method to analyze the hazard ratio (HR) of each primary predictor variable in the model. Because the TG/HDL-C ratio was not normally distributed in the dialysis patients, we constructed plots of the TG/HDL-C ratios and HRs of the outcomes using the Lowess function. The results revealed a nonlinear relationship, suggesting the need for stratification of the patients into quintiles according to their TG/HDL-C ratio for outcome analysis. Therefore, we stratified the patients into quintiles according to their TG/HDL-C ratio. The primary predictor variable was the TG/HDL-C ratio in each quintile. We also selected non-HDL-C (defined as T-CHO minus HDL-C)^27^ and TG/HDL-C ratio^5^ >3.8 as primary predictors according to published data. Similarly, because non-HDL-C was also not normally distributed, we reconstructed plots of non-HDL-C and HRs of the outcomes using the Lowess function and stratified the patients into tertiles accordingly for analysis.

The modification effects of nutritional status, inflammatory status, and diabetic status on the relationship between primary predictor variables and outcomes were also evaluated in predefined subgroups, which were defined according to the median values of GNRI and hs-CRP, or based on the patient's medical condition (presence of diabetes mellitus [DM]). The adjusted covariates included age, sex, dialysis vintage, dialysis modality, patient cohorts, presence of DM and hypertension, concurrent CV disease, GNRI, hs-CRP and calcium phosphate product (CaxP) levels in the analysis of a CV event, composite outcome, and all-cause mortality. To further evaluate and compare the predictive performance of the TG/HDL-C ratio and non-HDL-C, we used receiver operating characteristic (ROC) curves for censored data and the area under the ROC curve (AUC) as the criterion. All of the statistical analyses were performed using SPSS version 19.0 software (SPSS, Inc, Chicago, IL). A *P* value <0.05 was considered to be statistically significant.

## RESULTS

### Baseline Characteristics of the Participants

The baseline characteristics of all of the participants, the participants receiving HD or PD and participants in the TG/HDL-C quintiles are summarized in Tables 1 and 2. In general, the patients receiving PD were younger, had a lower percentage of diabetes, lower albumin levels, and higher T-CHO and LDL-C levels. However, similar levels of TG, HDL-C, TG/HDL-C ratio, and GNRI were found in the patients undergoing either HD or PD (Table 1). Overall, the patients in the TG/HDL-C quintiles had different percentages of diabetes, lipid profiles, hs-CRP level, and nutritional status (Table 2).

**TABLE 1:**
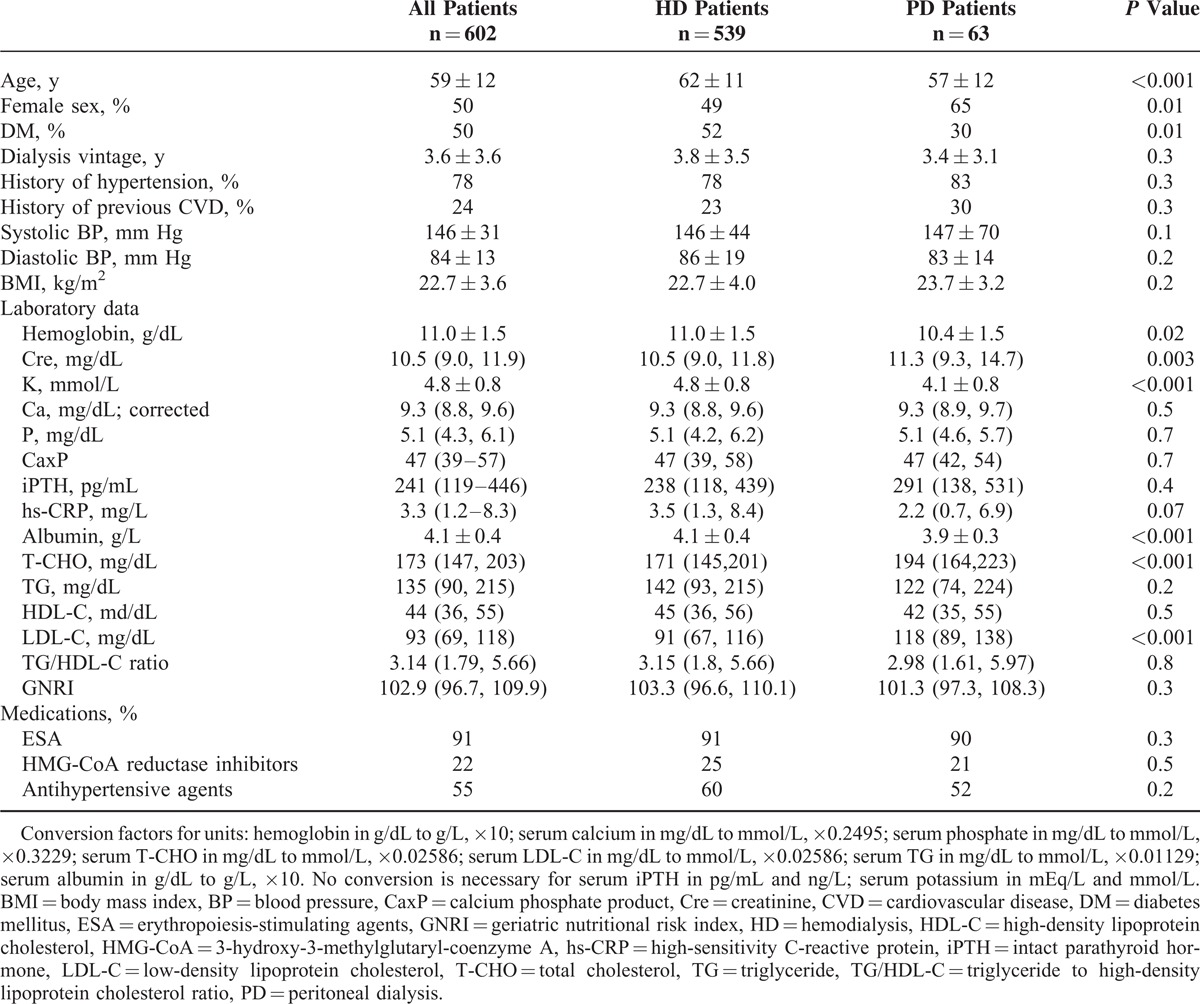
Baseline Characteristics of all Patients and the Patients Undergoing HD and PD

**TABLE 2:**
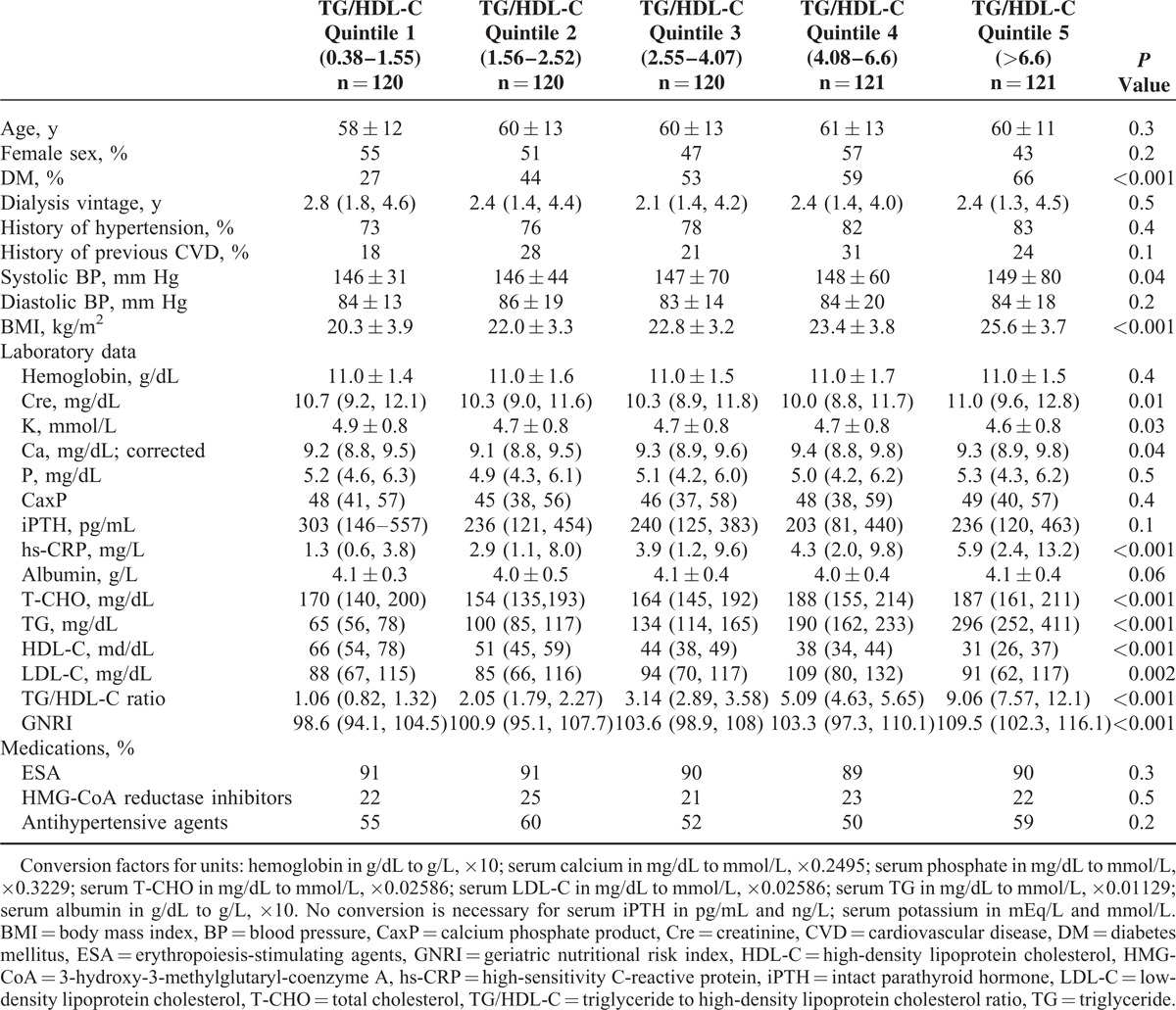
Baseline Characteristics of the Patients by TG/HDL-C Quintiles

### Outcomes

Overall, 203 of the participants experienced CV events, and 169 patients died, of whom 104 died due to CV events. Two hundred fifty-four patients reached the composite CV outcome. Of the 203 patients with CV events, 67 had intracranial hemorrhage or ischemic stroke, 106 had coronary artery disease (either nonfatal acute myocardial infarction or coronary revascularization/coronary bypass surgery), 24 had peripheral arterial occlusion disease, and 6 were hospitalized for heart failure. Of the 104 patients with CV death, 45 were due to coronary artery disease, 37 sudden cardiac death, 18 cerebrovascular events, and 4 were defined as arrhythmia. The median follow-up period was 3.9 years (range 0.2–7.4 years).

### Associations Between TG/HDL-C Ratio, Lipid Profiles, and Outcomes

In the multivariate Cox regression model, the patients with a higher TG/HDL-C level (quintile 5) had a higher incidence of CV events (adjusted HR 2.03, 95% confidence interval [CI] 1.19–3.47), CV mortality (adjusted HR 1.91, 95% CI 1.07–3.99), composite CV outcome (adjusted HR 2.2, 95% CI 1.37–3.55), and all-cause mortality (adjusted HR 1.94, 95% CI 1.1–3.39) compared with the patients in quintile 1. Taking TG/HDL-C ratio >3.8 as a dichotomous variable, the patients with a TG/HDL-C ratio >3.8 had a higher incidence of composite CV outcome. Similarly, the patients in non-HDL-C tertile 3 also had a higher incidence of CV events, CV mortality, and composite CV outcome compared with the patients in non-HDL-C tertile 1 (Table 3). Neither HDL-C nor TG level predicted all outcomes (all *P* > 0.05).

**TABLE 3:**
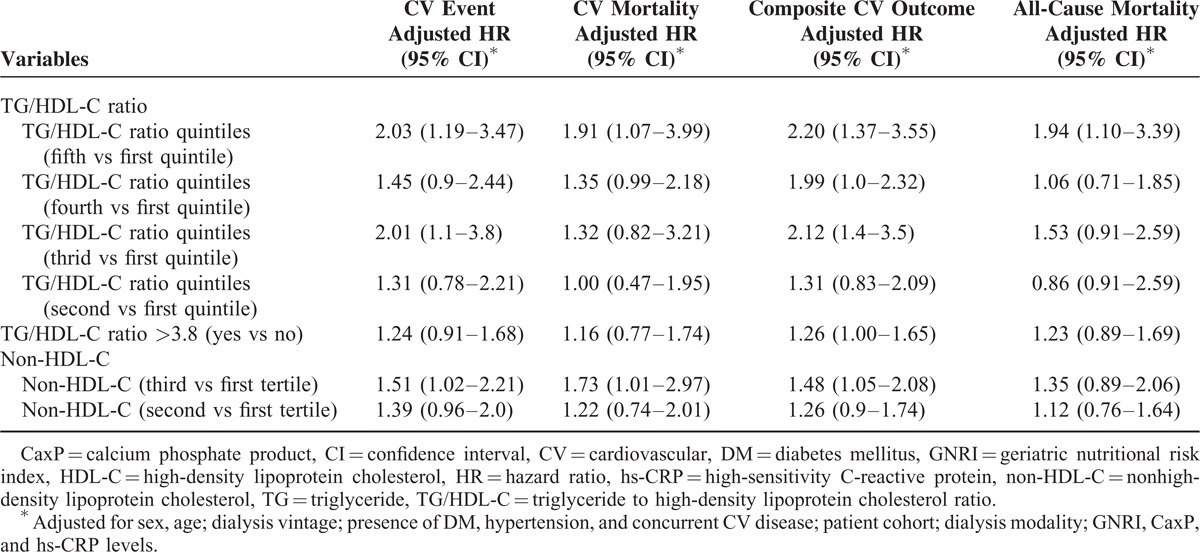
HRs of Atherogenic Dyslipidemia in Predicting the Outcomes Using Cox Proportional Hazards Regression Models With Multivariate Adjustments

The HRs of the 4 outcomes of the patients by respective quintile of TG/HDL-C ratio are shown in Table 3. As the TG/HDL-C level increased, the HRs of the composite CV outcomes also gradually increased (*P* for trend = 0.001), and there was an identical trend for the HRs of all-cause mortality (*P* for trend = 0.02). In addition, as the TG/HDL-C level increased, the HRs of CV events and CV mortality also gradually increased (*P* for trend = 0.007 and 0.04, respectively). In contrast, as the non-HDL-C level increased, the HRs of CV events, CV mortality, composite CV outcome, and all-cause mortality did not increase accordingly (*P* for trend = 0.09, 0.1, 0.08, and 0.4, respectively).

### Effect Modification of Prespecified Subgroups on the Relationship Between TG/HDL-C Ratio and Outcomes

**FIGURE 1:**
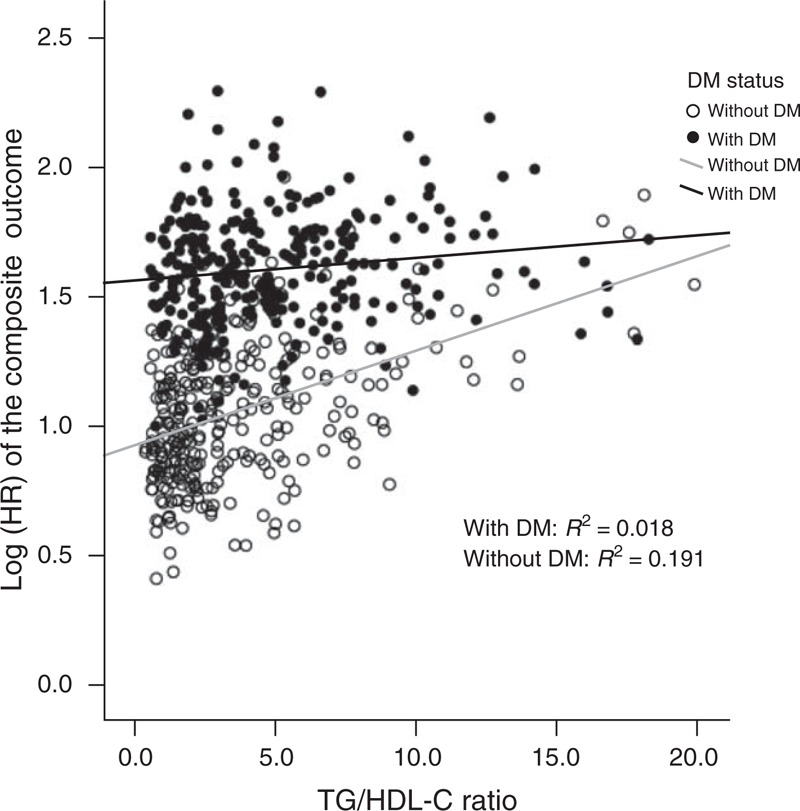
Association between the adjusted HRs of the composite CV outcomes (with log-transformation) and the TG/HDL-C ratio in dialysis patients with or without diabetes. In the diabetic dialysis patients, the TG/HDL-C ratio did not have an association with the composite outcome. CV = cardiovascular, DM = diabetes mellitus, HDL-C = high-density lipoprotein cholesterol, HR = hazard ratio, TG = triglyceride.

The predictive power of the TG/HDL-C ratio on the composite CV outcomes and all-cause mortality was consistent in all of the prespecified subgroups, regardless of their inflammatory condition (hs-CRP level) and nutritional status (GNRI level), except for DM status. We found an interaction between TG/HDL-C ratio and DM status in the prediction of the composite CV outcomes (*P* = 0.04, for interaction). As shown in Figure 1, in the patients without DM, the TG/HDL-C ratio had a significant association with the HRs of the composite CV outcomes; however, this association did not exist in the DM patients. In the Cox regression model, only nondiabetic patients with higher TG/HDL-C levels had a higher incidence of the composite CV outcome (*P* for trend = 0.03, adjusted HR 2.91, 95% CI 1.35–6.26, quintile 5 vs quintile 1) and CV events (*P* for trend = 0.007, adjusted HR 3.69, 95% CI 1.63–8.38, quintile 5 vs quintile 1).

### ROC Analysis of the TG/HDL-C Ratio and Non-HDL-C Level as Predictors of the Outcomes

**FIGURE 2:**
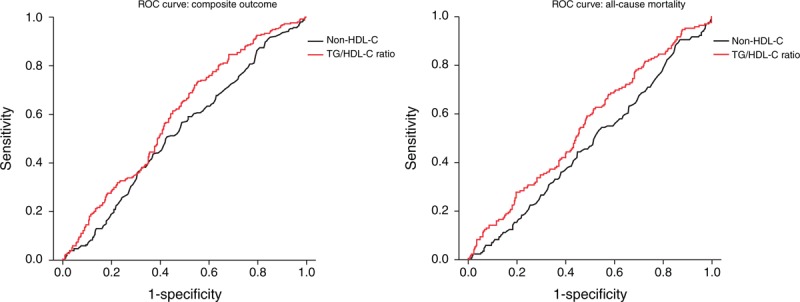
ROC curve of the TG/HDL-C ratio and non-HDL-C level as predictors of the outcomes. The TG/HDL-C ratio had a superior predictive performance for composite CV outcome and all-cause mortality (*P* = 0.01 and 0.02, respectively, by DeLong test). CV = cardiovascular, HDL-C = high-density lipoprotein cholesterol, ROC = receiver operating characteristic, TG = triglyceride.

The AUC for the TG/HDL-C ratio and non-HDL-C level versus outcomes are shown in Figure 2. The TG/HDL-C ratio and non-HDL-C levels had a similar predictive performance for CV outcome and CV death; however, the TG/HDL-C ratio had a superior predictive performance for composite CV outcome (AUC = 60.0, 95% CI 55.4–64.0 vs AUC = 52.8, 95% CI 55.2–48.2, *P* = 0.01 by DeLong test) and all-cause mortality (AUC = 55.3, 95% CI 52.7–61.0 vs AUC = 47.7, 95% CI 52.5–42.6, *P* = 0.02 by DeLong test).

## DISCUSSION

The main finding of this study is that the baseline TG/HDL-C ratio was predictive of composite CV outcomes and all-cause mortality in prevalent dialysis patients regardless of nutritional or inflammatory status. In addition, it had a noteworthy interaction with DM status when assessing the prediction of outcomes. Atherogenic dyslipidemia in dialysis patients with the TG/HDL-C ratio for stratification of its severity should be recognized as a crucial and easily available tool to predict long-term CV outcomes and survival.

Dialysis patients have unique lipid profiles, and the association between lipid profiles and CV outcomes and survival are completely different from those in the general population.^19^ In several landmark studies, dialysis patients have been reported to have unique correlations between levels of T-CHO and LDL-C, and CV outcomes, which is known as the “cholesterol paradox.”^28,29^ In addition, recent published data have shown the lack of an association between HDL-C levels and the risk of CV events in diabetic HD patients,^22^ and in a large HD cohort who were followed up for 3 years, HDL-C and TG did not predict CV or all-cause mortality in HD patients.^19^ In the current study, we used the TG/HDL-C ratio to evaluate long-term CV composite outcomes and survival in both HD and PD patients, and the results clearly demonstrated their predictive power (Table 3). To the best of our knowledge, this is the first study to demonstrate the predictive ability of the TG/HDL-C ratio for CV outcomes and survival in patients undergoing prevalent dialysis. The TG/HDL-C ratio may therefore be an optimal marker to predict CV outcomes in dialysis patients, as it has already been utilized in the general population.^1,3,7,9^ In our results, a TG/HDL-C ratio >3.8 also predicted the occurrence of composite CV events, which could be partially explained by reports that a TG/HDL-C ratio >3.8 is a strong indicator of the presence of atherogenic small LDLs.^5^ In other words, a higher TG/HDL-C ratio indicates a milieu with abundant atherogenic lipoprotein particles resulting in consequent CV complications not only in the general population but also in dialysis patients. In addition, the non-HDL-C level predicted the occurrence of CV events, which is consistent with the results of previous studies.^27,30^

In women at high risk of CV events, the TG/HDL-C ratio was a powerful predictor of coronary artery disease and all-cause mortality, with an approximately 2-fold increase after 6 years of follow-up.^10^ Furthermore, other studies have shown modest to strong effect sizes (adjusted HR from 2 to 16) in the prediction of CV events and mortality.^7,8^ In our results, the effect size of the TG/HDL-C ratio in the prediction of composite CV outcome was moderate (adjusted HR 2.2, Table 3); however, the effect sizes were attenuated in CV and all-cause mortality (Table 3). A possible explanation is that malnutrition–inflammation complex syndrome interacts with atherogenic lipoprotein particles in dialysis patients,^28^ and nutritional and inflammatory status are independently associated with CV outcomes and survival.^26,31,32^ The full adjustment for hs-CRP and GNRI levels in our analysis may have attenuated the effect sizes. In addition, CV and all-cause mortality in dialysis patients are attributable to not only atherosclerotic vascular events but also vascular calcification.^33^ In our fully adjusted model, we also included CaxP to adjust the effects from vascular calcification, which would attenuate the effect size further.

We found an interaction between the TG/HDL-C ratio and DM status in the prediction of outcomes (Figure 1) and as the baseline characteristics show, the prevalence of DM increased across the TG/HDL-C ratio quintiles (Table 2). This finding is consistent with that in the general population, and it has been reported that patients with a high TG/HDL-C ratio are predisposed to have metabolic syndrome or consequent DM.^12,34^ In other words, the patients with a high TG/HDL-C ratio, metabolic syndrome, and DM may be considered to have a metabolic continuum, of which diabetes is the end stage. In patients with both DM and atherogenic dyslipidemia, diabetic status is crucial when assessing CV outcomes compared with atherogenic dyslipidemia. As shown in landmark trials,^12,13,35^ the manipulation of high TG and low HDL-C levels in DM patients with medical interventions does not reduce the risk of CV outcomes. Consequently, the utility of the TG/HDL-C ratio for the prediction of long-term CV outcomes in diabetic dialysis patients should be carefully explored in further large-scale investigations.

The strengths of this study are its prospective nature and full adjustment for multiple CV and survival predictors, especially inflammatory (hs-CRP) and nutritional (GNRI) factors, in the outcome analysis. However, there are some limitations to this study. First, the observational nature of this study precludes the conclusions of a causal relationship. Second, we did not check the specific lipoproteins related to atherogenic dyslipidemia such as apolipoprotein B, which may be more specific to LDL particles. In addition, the relationship between TG/HDL-C ratio and apolipoproteins in dialysis patients needs to be validated further. Third, this was a single-center study, and all of the participants were treated by the same physicians and underwent uniform laboratory measurements during the observation period, which guaranteed the accuracy of our results. However, our conclusions cannot be generalized to other ethnicities.

In summary, our results suggest that the TG/HDL-C ratio can independently predict composite CV outcomes and mortality in prevalent dialysis patients, especially nondiabetic dialysis patients.
